# Retraction: DEAD-Box Helicase 5 Interacts With Transcription Factor 12 and Promotes the Progression of Osteosarcoma by Stimulating Cell Cycle Progression

**DOI:** 10.3389/fphar.2019.01657

**Published:** 2019-12-23

**Authors:** Frontiers Editorial Office

Following publication, concerns were raised regarding the scientific validity of the article. An investigation was therefore conducted in accordance with our established procedures. As the raw data presented in the manuscript could not be provided, the Specialty Chief Editor concluded that the conclusions and assertions could not be sufficiently supported by the material provided.

The retraction of the article was approved by the Chief Editors of Pharmacology and the Editor-in-Chief of Frontiers.

The authors agree to the retraction.

